# Advancing the Role of Proton Therapy for Spine Metastases Through Diagnostic Scan–Based Planning

**DOI:** 10.14338/IJPT-23-00005.1

**Published:** 2023-11-08

**Authors:** Cameron W. Swanick, Michael H. Shang, Kevin Erhart, Jonathan Cabrera, James Burkavage, Tomas Dvorak, Naren Ramakrishna, Zhiqiu Li, Amish Shah, Sanford L. Meeks, Omar A. Zeidan, Patrick Kelly

**Affiliations:** 1 Center for Advanced Radiation Therapy, Orlando Health Cancer Institute, Orlando, FL, USA; 2.decimal, LLC, Sanford, FL, USA; 3 Varian Medical Systems, Inc., Palo Alto, CA, USA

**Keywords:** metastases, palliative, spine

## Abstract

**Purpose:**

Many patients with metastatic cancer live years beyond diagnosis, and there remains a need to improve the therapeutic ratio of metastasis-directed radiation for these patients. This study aimed to assess a process for delivering cost-effective palliative proton therapy to the spine using diagnostic scan–based planning (DSBP) and prefabricated treatment delivery devices.

**Materials and Methods:**

We designed and characterized a reusable proton aperture system that adjusts to multiple lengths for spine treatment. Next, we retrospectively identified 10 patients scan treated with thoracic proton therapy who also had a diagnostic computed tomography within 4 months of simulation. We contoured a T6-T9 target volume on both the diagnostic scans (DS) and simulation scans (SS). Using the aperture system, we generated proton plans on the DS using a posterior–anterior beam with no custom range compensator to treat T6-T9 to 8 Gy × 1. Plans were transferred to the SS to compare coverage and normal tissue doses, followed by robustness analysis. Finally, we compared normal tissue doses and costs between proton and photon plans. Results were compared using the Wilcoxon signed-rank test.

**Results:**

Median D_95%_ on the DS plans was 101% (range, 100%–102%) of the prescription dose. Median D_max_ was 107% (range, 105%–108%). When transferred to SS, coverage and hot spots remained acceptable for all cases. Heart and esophagus doses did not vary between the DS and SS proton plans (*P* >.2). Robustness analysis with 5 mm X/Y/Z shifts showed acceptable coverage (D_95%_ > 98%) for all cases. Compared with the proton plans, the mean heart dose was higher for both anterior–posterior/posterior–anterior and volumetric modulated arc therapy plans (*P* < .01). Cost for proton DSBP was comparable to more commonly used photon regimens.

**Conclusion:**

Proton DSBP is technically feasible and robust, with superior sparing of the heart compared with photons. Eliminating simulation and custom devices increases the value of this approach in carefully selected patients.

## Introduction

Many patients with metastatic cancer are living years beyond their diagnosis, with recent data for metastatic breast cancer demonstrating 5-year survival rates of 30% to 40% [1, 2]. These numbers will likely continue to improve for many malignancies with the expanded use of targeted therapies [3–5] and immunotherapies [6]. At the same time, the role of radiation therapy for definitive treatment of metastatic sites has been called into question, and recently released results of the NRG BR-002 trial failed to demonstrate improvement in progression-free or overall survival with the addition of ablative local therapy to metastatic sites in breast cancer patients [7]. These results will likely lead insurers to decline stereotactic body radiation therapy coverage for breast cancer metastases.

With stereotactic body radiation therapy unavailable, many cancer patients will receive palliative radiation with less precise 2- or 3-field photon techniques, often with associated toxicity, including nausea and diarrhea in the short term [8, 9] and potentially pneumonitis and cardiac events in the long term [10, 11]. Furthermore, the frequency and severity of these adverse events may be amplified by systemic therapies [12–14], and for many newer drugs, the safety of concurrent radiation delivery is not well-established.

One way to reduce the dose to normal tissues from palliative radiation therapy is to use proton therapy. This technique is especially well-suited for treating spine metastases as dose to anterior structures can be essentially eliminated. Historically, proton therapy has not been used in the palliative setting because of the inherent cost and time needed for planning. It has been previously demonstrated that radiation treatment planning using a patient’s existing diagnostic scans in lieu of a formal simulation scan (a process known as diagnostic scan-based planning [DSBP]) can increase efficiency of photon-based treatment planning [15]. To our knowledge, this process has not yet been assessed for passive-scatter proton therapy, a technique that carries the added complexity of patient-specific treatment delivery devices (eg, apertures and compensators) that contribute to planning time and cost. Therefore, the current study aimed to assess a process for delivering efficient and cost-effective palliative proton therapy by using a combination of DSBP and prefabricated treatment delivery devices.

## Materials and Methods

### Aperture System

**Figure 1.**
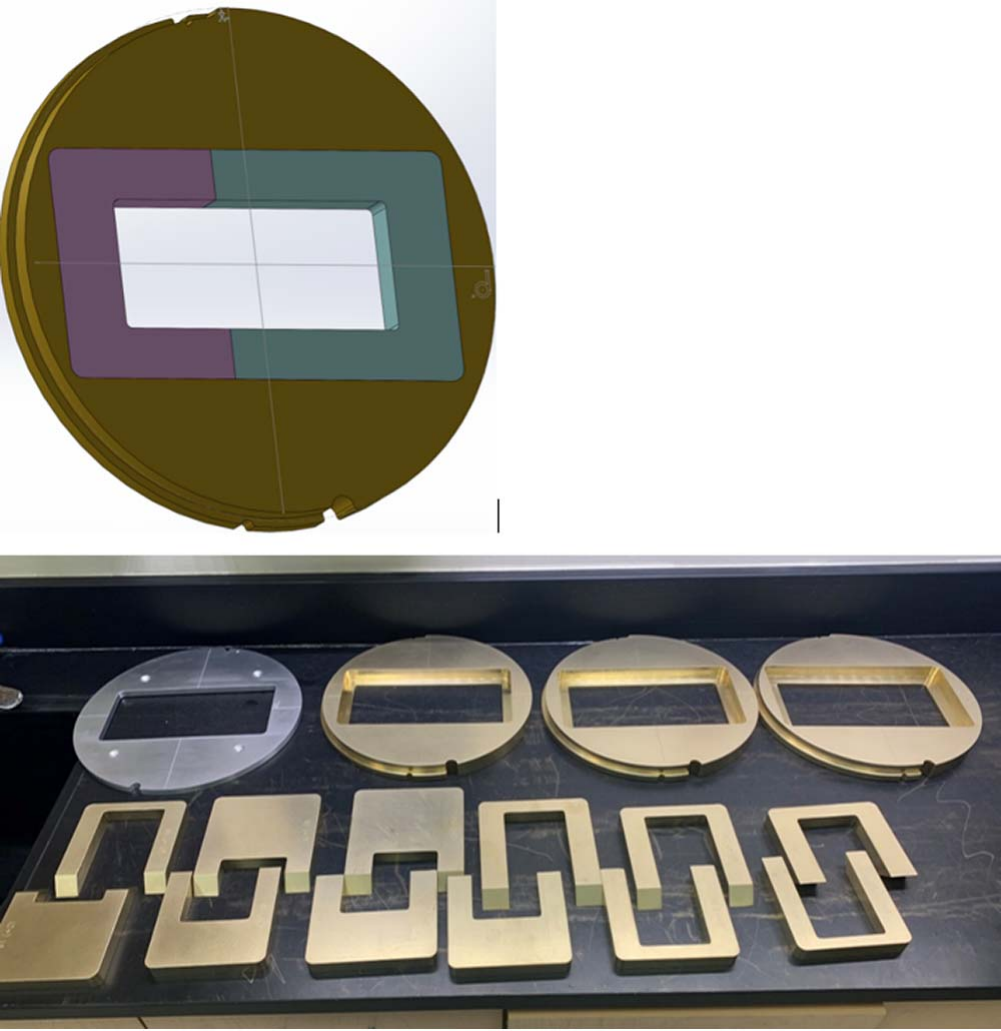
Computer-aided design rendering (top) and actual photo (bottom) of the reusable proton aperture plug-and-base system capable of adjusting to multiple field sizes for spine treatment. The design comprises an inner fixed opening with 2 inserts (lavender and blue). For this set of apertures, the width is fixed at 7 cm, and optional lengths range from 8 to 20 cm, depending on the combination of inserts used.

For the first part of this study, we designed, built, and characterized a reusable proton aperture system capable of adjusting to multiple field sizes for thoracic spine treatment. We designed a series of interchangeable inserts with a fixed aperture width of 7 cm and optional lengths of 8, 12, 16, and 20 cm. We selected a 7-cm fixed-width insert based on average thoracic vertebrae size and confirmed at the time of planning (detailed below) that this width would allow for the inclusion of the entire vertebrae within the aperture for all cases. Optional lengths were then selected based on common clinical scenarios and average vertebrae heights; for example, an 8-cm length allows for coverage of 4 vertebrae with the 95% isodose line. The maximum length was limited to 20 cm, which was the longest that could be achieved while still allowing enough mating surface to physically secure the aperture system to the snout. The aperture system was designed so that the different aperture lengths could be assembled with 2 prespecified inserts overlapping with slanted surfaces at the junction to minimize leakage (**Figure 1**). Once in place, the inserts are secured with the aperture ring.

Once the design was finalized, .decimal fabricated the prototype plug-and-base aperture system for dosimetric characterization and radiation leakage testing. Leakage testing was performed using radiochromic films placed on blank apertures to ensure that the interlocking between the base plate apertures and the inserts was sufficiently tight to prevent any leakage of protons through the seams. The results demonstrated that the prototype system was as effective in blocking radiation as clinical apertures.

A preliminary clinical treatment workflow was tested using an anthropomorphic thoracic phantom. We demonstrated that it is possible to acquire posterior–anterior (PA) X-rays through the devices while the patient is in the treatment position. The process is similar to acquiring a conventional port film on a linear accelerator. First, a wide-open PA image is obtained without the inserts to allow for visualization of the surrounding anatomy to confirm vertebral body level. We then place the inserts and obtain the smaller image defined by the treatment aperture.

### Case Selection and Treatment Planning

For the second part of this study, we retrospectively identified 10 patients previously treated with proton therapy for thoracic malignancies who also had a diagnostic computed tomography (CT; either dedicated CT or positron-emission tomography/CT) of the thorax within 4 months of the simulation. The relevant diagnostic and simulation scans were exported from the treatment planning system, anonymized, and reimported for the selected cases. The diagnostic CT scans were then corrected for rotation, roll, and pitch to simulate a flat, head-first, supine treatment position. The magnitude of geometric corrections (translational and rotational) was different for every case based on the manual fusion of the diagnostic scans (DS) image with the simulation scans (SS) image. The spine anatomy was used as the basis for fusion. For each case, 1 physician (CWS) contoured T6-T9 as the target volume on both the diagnostic and simulation scans.

Respecting the parameters of the reusable proton aperture system described above, we generated passive-scatter proton plans on each patient’s diagnostic scan using a single PA beam with no custom range compensator. The target was T6-T9, inclusive, and the dose was 8 Gy × 1. The dose was prescribed such that at least 95% of the target (D_95%_) received 100% of the dose. We employed our own dose range calculation uncertainty criteria of 3% + 3 mm on the DS image dataset to develop an appropriate beam range for each case. When this range was used on the SS image dataset, we noticed that the target was appropriately covered for all cases. Therefore, moving forward, we used the same 3% + 3–mm criteria on the DS images without including this criterion in the robustness analysis (detailed below) because the main clinical concern is setup uncertainty.

All plans were evaluated by a physician and found to be clinically acceptable. The approved treatment plans were then transferred to the corresponding simulation scans using 2-dimensional mid-plane projection to model treatment setup and delivery. We then compared target coverage, dose heterogeneity, and doses to normal tissues, specifically mean and maximum heart dose and mean and maximum esophagus dose. Finally, to provide a dosimetric comparison to a commonly used palliative treatment approach, we created an anterior–posterior/PA (AP/PA) photon plan on the simulation scan for each case and recorded the same dosimetric data points described above. In addition, we created a volumetric modulated arc therapy (VMAT) plan (also on the simulation scan) for each case to assess organs at risk (OARs) sparing compared with the proton plans. Results were compared using the Wilcoxon signed-rank test.

### Robustness Analysis

We performed robustness analysis with ± 5 mm X, Y, and Z shifts for each passive-scatter proton plan transferred from the diagnostic scan to the simulation scan. For each subplan (a total of 6 plans per patient), we recorded the D_95%_ and D_max_, with the primary goal of screening for outliers that would not be clinically acceptable.

### Financial Analysis

To assess the cost-efficiency of the process described above, we looked at technical and professional costs associated with single-fraction proton treatment without the need for simulation or custom treatment delivery devices. This was then compared with single-fraction photon treatment without simulation (essentially modeling cost for a DSBP approach for photons) and the more common palliative regimen of 10-fraction 2-field photon treatment with simulation. For completeness, we also assessed single-fraction VMAT costs with and without simulation. All costs were calculated using Centers for Medicare & Medicaid Services reimbursement rates.

## Results

### Coverage and Heterogeneity

**Figure 2.**
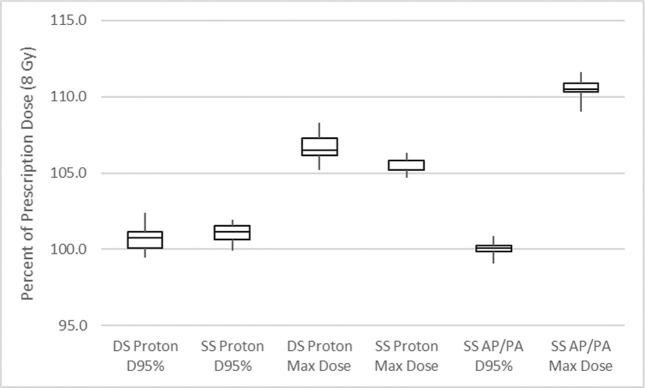
Box and whisker plot for target D95% coverage and max dose on diagnostic scan (DS) proton plans, simulation scan (SS) proton plans, and SS photon anterior–posterior/posterior–anterior (AP/PA) plans.

**Figure 3.**
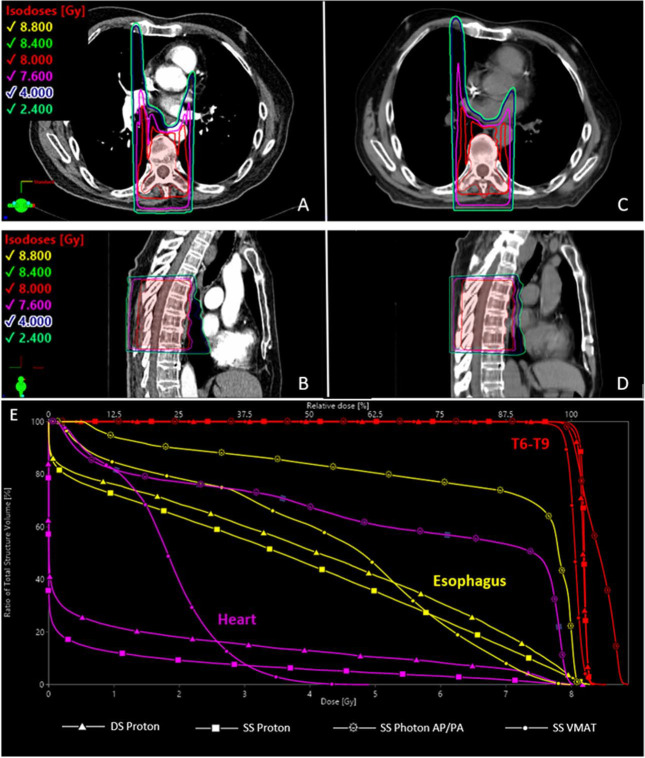
Example case for passive-scatter proton plan generated on diagnostic scan (DS) (panels A [axial view] and B [sagittal view]) and then transferred to simulation scan (SS) (panels C [axial view] and D [sagittal view]). Each plan used a single posterior–anterior (PA) beam with a 12 cm (L) × 7 cm (W) aperture and no custom range compensator. The dose-volume histogram for the DS proton plan, the SS proton plan, the SS photon anterior–posterior (AP)/PA plan, and the volumetric modulated arc therapy (VMAT) plan for this patient’s case is depicted in panel E.

For the DS passive-scatter proton plans, the median D_95%_ was 101% (range, 100%–102%) of the prescription dose (8 Gy) using the same 12 cm (L) × 7 cm (W) aperture for each case. The median D_max_ was 107% (range, 105%–108%). When the plans were transferred to the simulation scans, coverage remained excellent and hot spots were acceptable for all cases with a median D_95%_ of 101% (range, 100%–102%) and a median D_max_ of 106% (range, 105%–106%). The comparison AP/PA photon plans resulted in similar coverage to the proton plans but demonstrated a clinically meaningful increase in dose heterogeneity with a median D_max_ of 110% (range, 109%–112%) (**Figure 2**). Representative axial and sagittal images of the proton plans from an example case are presented in **Figure 3**.

### Organ at Risk Doses

OAR doses across techniques are presented in **Table 1**. Heart and esophagus mean and maximum doses did not vary significantly between the diagnostic and simulation scan proton plans (*P* > .2 for all). Compared with the proton plans, the mean heart dose was significantly higher for the AP/PA photon plans (median 4.7 Gy vs 0.4 Gy, *P* < .01) and VMAT plans (median 1.8 Gy vs 0.4 Gy, *P* < .01). Compared with proton plans, mean esophagus dose was also significantly higher for the AP/PA photons plans (median 7.1 Gy vs 4.9 Gy, *P* < .01) but not significantly different for VMAT plans (median 4.8 Gy vs 4.9 Gy, *P* > .05). Heart and esophagus max doses were not significantly different between the proton and photon plans (*P* > .05); however, heart and esophagus max doses were lower for VMAT plans compared with proton plans (*P* < .05 for both).

**Table 1.** Dosimetry comparison of median OAR doses across techniques.

Metric	DS proton	SS proton	SS AP/PA	SS VMAT
Heart mean dose (Gy), Median (range)	0.4 (0.1–1.1)	0.4 (0.1–0.5)	4.7 (2.3–6.6)	1.8 (0.9–3.0)
Heart max dose (Gy), Median (range)	8.2 (5.8–8.4)	8.1 (7.3–8.4)	8.2 (8.0–8.4)	5.5 (4.1–8.3)
Esophagus mean dose (Gy), Median (range)	3.7 (0.4–6.2)	4.9 (2.0–5.5)	7.1 (5.3–7.6)	4.8 (4.1–5.6)
Esophagus max dose (Gy), Median (range)	8.3 (3.4–8.4)	8.4 (7.4–8.4)	8.2 (8.0–8.3)	8.2 (6.7–8.4)

**Abbreviations:** OAR, organ at risk; DS, diagnostic scan; SS, simulation scan; AP/PA, anterior–posterior/posterior–anterior; VMAT, volumetric modulated arc therapy.

### Robustness Analysis

**Figure 4.**
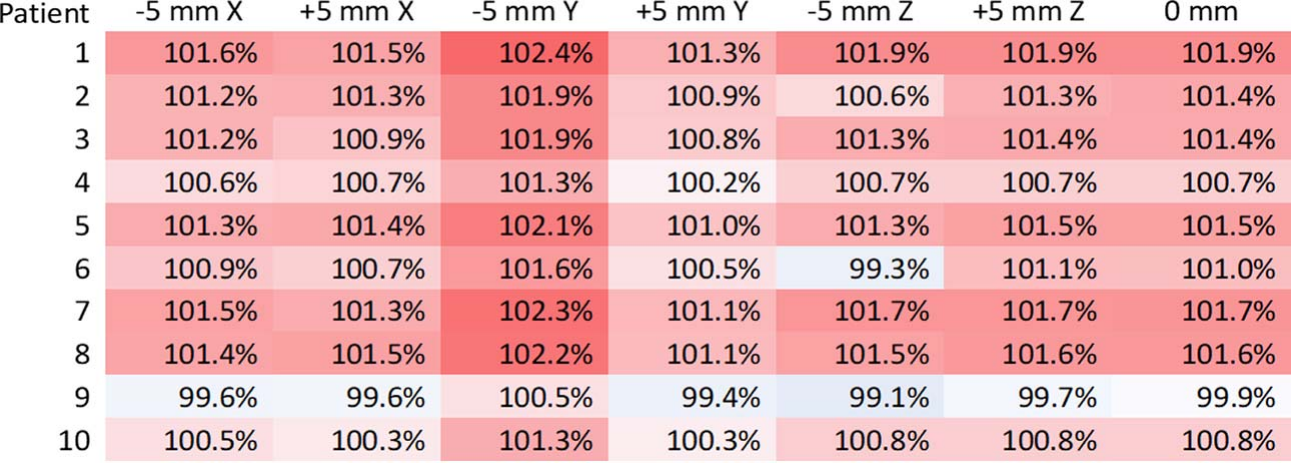
Color-coded heat map table for robustness analysis for target D95% coverage for passive-scatter proton plans generated on the diagnostic scan and transferred to simulation scan. Percentages are relative to the prescription dose of 8 Gy in 1 fraction.

Robustness analysis with ± 5 mm X, Y, and Z shifts showed that coverage remained clinically acceptable with D_95%_ > 98% for all cases with no outliers. A heat map depicting D_95%_ coverage for each passive-scatter proton subplan is presented in **Figure 4**.

### Financial Analysis

For single-fraction proton treatment without simulation or custom devices, we calculated a technical cost of $3151.18 and a professional cost of $705.63 for a total cost per patient of $3856.81. By contrast, a single-fraction photon regimen without simulation was $1895.99, and a 10-fraction photon regimen with simulation was $4364.17 (**Table 2**). Single-fraction VMAT treatment without simulation was similar to our proton approach, with a total cost of $3442.45.

**Table 2.** Cost data estimated from Centers for Medicare and Medicaid Services reimbursement rates.

Technique	Technical	Professional	Total
Proton, no sim, no devices, 1 fraction	$ 3,151.18	$ 705.63	$ 3,856.81
Photon 2D, no sim, 1 fraction	$ 1,246.70	$ 649.29	$ 1,895.99
Photon 2D, with sim, 10 fractions	$ 3,430.92	$ 933.25	$ 4,364.17
Photon VMAT, no sim, 1 fraction	$ 2,544.56	$ 897.89	$ 3,442.45
Photon VMAT, with sim, 1 fraction	$ 2,544.56	$ 982.48	$ 3,527.04

**Abbreviations:** 2D, 2-dimensional; VMAT, volumetric modulated arc therapy.

## Discussion

In this study, we demonstrated that using a patient’s existing imaging and prefabricated treatment devices can generate a clinically acceptable and robust palliative proton treatment plan with significantly decreased normal tissue doses compared with a commonly used photon regimen. This proof of concept is an important first step toward expanding the use of proton therapy in the palliative setting, particularly for patients expected to live years beyond their diagnosis.

We also found that using our approach for a single-fraction proton therapy treatment course was less costly than the commonly used 10-fraction photon course and only slightly more costly than a single-fraction VMAT course. Cost is arguably the most significant barrier to using proton therapy in this setting, and the cost savings from eliminating simulation and custom treatment devices increases the overall value of this approach in carefully selected patients.

Proton therapy is an especially attractive option for the palliative treatment of breast cancer patients, as 5-year survival rates for de novo metastatic breast cancer are similar to those for malignancies more commonly treated with definitive proton therapy, such as locally advanced lung cancer, hepatobiliary cancers, and primary brain tumors [16–18]. In addition, patients with metastatic breast cancer frequently receive treatment with novel systemic therapies, many of which are known to potentiate the toxicities of radiation therapy [13, 19]. With stereotactic body radiation therapy likely falling out of favor for these patients due to the results of NRG BR-002, exploring novel strategies to reduce normal tissue doses in this population is warranted.

Multiple studies have suggested that the risk of cardiac death increases with mean heart dose [10, 11, 20, 21], and this risk is often realized within just a few years of treatment. In a retrospective study of over 748 patients treated with radiation therapy for non–small cell lung carcinoma, Atkins et al [20] found that a mean heart dose of ≥ 10 Gy was associated with a significantly increased risk of a major cardiac adverse event in patients with no preexisting coronary artery disease, and this difference was detected with a median follow up of just 20.4 months. Of note, the Atkins study included patients treated with standard fractionation, and therefore, a mean heart dose of 10 Gy translated to a biologically effective dose of 11 Gy (assuming 30 fractions of treatment with an alpha/beta of ∼3 for the heart [22]). This would compare with a biologically effective 12.1-Gy heart dose for the cases in this study treated with a single fraction of AP/PA photons, suggesting that patients treated with this approach may be at significant risk for short-term cardiac complications. By contrast, the proton plans in this study delivered a median mean heart dose of 0.4 Gy or a biologically effective dose of 0.5 Gy.

In addition to long-term toxicity from large, single-fraction doses, short-term toxicities, such as nausea and vomiting, are common in patients receiving palliative treatment near the upper abdomen [23, 24]. In our practice, physicians often choose 5- or 10-fraction palliative regimens for patients requiring large treatment fields to reduce the likelihood of gastrointestinal adverse effects. In the current study, we evaluated the dose to the esophagus as this was the relevant OAR for the study target (T6-T9) and found that mean esophagus dose was markedly higher with AP/PA photon plans versus the corresponding proton plan. Of note, the stomach dose was not assessed in our study as it was not a relevant OAR, but other studies have clearly demonstrated the benefit of proton therapy in reducing acute nausea and vomiting in patients receiving spine treatment [25].

Our study builds on previous work that demonstrated the feasibility of DSBP for treating critically ill patients with emergent photon radiation therapy [15], an approach that we are now using in our clinic for appropriately selected patients. The current proton-based approach offers a few advantages over the photon-based approach. First, as previously discussed, the dose to normal tissues and attendant side effects are expected to be less. In addition, proton-based planning with a single PA beam would be more robust in patient position and anatomy changes than photon planning with multiple beams. For example, in the time between the diagnostic scan used for planning and the time of treatment, a patient could experience a weight change, develop ascites or abdominal distention, all of which could change attenuation and affect coverage for a multifield photon plan but would not significantly affect coverage from a single PA proton beam. Finally, we expect to see increased craniospinal treatment for patients with leptomeningeal disease now that randomized data demonstrate improved survival in patients with breast and lung cancer [26]. Although our study addresses treatment to a fraction of the spinal column, we hope to apply the same concept to craniospinal treatment of symptomatic inpatients for whom treatment must be expedited.

Our study had several limitations. We only assessed a small portion of the spinal column, and additional studies will be needed to ensure that this methodology can be safely applied in the cervical and lumbosacral regions. However, we purposely selected the thoracic spine levels with the greatest kyphosis to ensure that distal coverage of the target over a 12-cm length could be achieved without a custom compensator, which was in fact the case for these patients. An additional limitation of our study is the small sample size of 10 patients, although we did determine that for this group of 10 consecutive clinical cases, there were no outliers in terms of acceptable target coverage or dose heterogeneity. We also recognize that without a custom range compensator, the distal edge of the beam is nonconformal, and in some cases, the distal edge of the beam ranges into the adjacent OARs. Although there are certainly clinical settings in which the biological consequences of the elevated linear energy transfer at the distal edge must be carefully considered, we do not think it is particularly relevant for single-dose palliative treatment with 8 Gy.

Of note, our study examined only passive-scatter proton therapy, as this technology is currently available for clinical use at our institution. We acknowledge that many modern proton therapy centers offer pencil beam scanning (PBS) only and that clinically acceptable plans can be generated using a single PA PBS beam. In fact, we would expect PBS plans to be more conformal than the passive-scatter plans presented here, and there would be no need for an aperture at all. However, PBS would require time for planning optimization followed by patient-specific quality assurance, thereby reducing the overall efficiency of the process. This study aimed to establish an efficient, broadly applicable approach that we believe is more readily achieved with passive-scatter planning.

Palliative proton DSBP is technically feasible and robust, and the superior sparing of normal tissues compared with standard photon plans can reduce the risk of short- and long-term toxicity. The careful elimination of cost-related barriers associated with proton therapy could allow clinicians to leverage the dosimetric advantages of this technique to improve the therapeutic ratio for palliative treatment of patients expected to live years after their treatment.
